# Supplementary material for: Adverse Cardiovascular Events in Non-Traumatic Intracranial Hemorrhage and Ischemic Stroke Survivors
Source: J Clin Med. 2022 Nov 22;11(23):6885. doi: 10.3390/jcm11236885 (PMC9739641; doi:10.3390/jcm11236885)
Supplement: Supplementary file 1 [file jcm-11-06885-s001.zip › jcm-2005945-supplementary.pdf]

Journal of
Clinical Medicine

MDPI

Supplementary Materials

Statistical analyses for propensity score matching.

The propensity score included cardiovascular risk factors and non-cardiovascular comorbidities from the baseline characteristics listed in table 1, namely age, sex, obesity, history of hypertension, diabetes, dyslipidemia, smoking, alcohol related diagnoses, chronic kidney disease, lung disease, chronic obstructive pulmonary disease, sleep apnea syndrome, liver disease, gastroesophageal reflux, thyroid diseases, anemia, inflammatory disease, cognitive impairment, poor nutrition, and illicit drug use. For every patient with history of non-traumatic intracranial hemorrhage, a propensity score-matched patient with ischemic stroke was identified and selected with the one-to-one nearest neighbor method (with a caliper of 0.01 of the standard deviation (SD) of the propensity score on the logit scale) and no replacement. The distributions of demographic data and comorbidities in the two cohorts was assessed with standardized differences, which were calculated as the difference in the means or proportions of a variable divided by a pooled estimate of the SD of that variable with 5% or less indicating a negligible difference between the means of the two cohorts (supplemental Figure S1 and S2).

Table S1. International Classification of Disease 10 (ICD-10) codes.

Comorbidity or Medical History	Existing Diagnoses
AF management care	I48
Strokes	
Ischaemic stroke	I63, I66, I67
Stroke, unspecified	I64
Haemorrhagic stroke	I60–I62, I69
Transient ischaemic attack	G45
Systemic embolism	I74.2–I74.9
Haemorrhages	
Intracranial bleeding	I60, I61, I62, S06.4, S06.5, S06.6
Major bleeding	I60, I61, I62, S06.4, S06.5, S06.6<br>D62, D683, D698, D699, H113, H922, J942, K661, K762, R040, R041, R042, R048, R049, R58, S271, T792<br>H313, H356, H431, H450, I230, I312, I600, I601, I602, I603, I604, I605, I606, I607, I608, I609, I620, I621, M250, S064, S065, S066, S260<br>I850, I983, K226, K250, K252, K254, K256, K260, K262, K264, K266, K270, K272, K274, K276, K280, K282, K284, K286, K290, K625, K920, K921, K922<br>N020, N021, N022, N023, N024, N025, N026, N027, N028, N029, N421, N920, N921, N923, N924, N930, N938, N939, N950, R31<br>Z513<br>(CCAM codes: FELF008, FELF011, FELF001, FELF004)
Coronary artery disease	I20–I25
Heart failure	I50, I110, I130, I132, I131, I139
Cardiac dysrhythmia	I47, I490–I493
Abnormal cardiac conduction	I44, I45, I494, I495, Z450, Z950
Valvular disease	I05–I091, I33–I39, Q22, Q23
Mitral stenosis	I342, I050, I052, Q232
Hypertension	I10–I15
Diabetes mellitus	E10–E14
Myocardial infarction	I21, I252
Peripheral arterial disease	I70–I73

Occlusions	I65, I77
Obesity	E65–E66
Abnormal renal function	N17–N19 (+N28) codes for renal insufficiency, transplantation (Z940, T861) and dialysis (Z49, Z992), E102, I12, I13
Liver disease	K70–K77, procedures for liver transplantation or resection
Dyslipidaemia	E78
Thyroid disease	E00–E07
Anaemia	D50–D64
Platelet or coagulation defect	D65–D69
Lung disease	J40–J70, J961
Including emphysema and chronic obstructive pulmonary disease	J43, J44
Alcohol-related diagnoses	E244, F10, G312, G621, G721, I426, K292, K70, K860, O354, P043, Q860, T51, Y90, Y91, Z502, Z714
Dementia	F00–F03
Accidental falls	W00–W19, R26
Cancer within preceding 5 years	Entire C-series
Inflammatory diseases	M05–M14, M45, M46, K50–K52, K81, K85
Digestive conditions	Entire K-series
Rheumatology	Entire M-series
Ophthalmology	Entire H-series
Pulmonology	Entire J-series

Table S2. Baseline characteristics of matched patients with no history of atrial fibrillation at baseline in ischemic stroke and on-traumatic intracranial hemorrhage survivors.

	**Ischemic Stroke**	**Non-Traumatic ICrH**	*p*	**Total**
	**(*n* = 11,873)**	**(*n* = 11,873)**		**(*n* = 23,746)**
Age (years), mean±SD	69.7±16.4	69.2±16.5	0.04	69.5±16.4
Sex (male), *n* (%)	6170 (52.0)	6351 (53.5)	0.02	12521 (52.7)
Hypertension, *n* (%)	6497 (54.7)	6634 (55.9)	0.07	13131 (55.3)
Diabetes mellitus, *n* (%)	1927 (16.2)	1991 (16.8)	0.26	3918 (16.5)
Smoker, *n* (%)	996 (8.4)	1119 (9.4)	0.01	2115 (8.9)
Dyslipidemia, *n* (%)	2008 (16.9)	2017 (17.0)	0.88	4025 (17.0)
Obesity, *n* (%)	896 (7.5)	962 (8.1)	0.11	1858 (7.8)
Heart failure, *n* (%)	1669 (14.1)	1314 (11.1)	<0.0001	2983 (12.6)
History of pulmonary edema, *n* (%)	151 (1.3)	177 (1.5)	0.15	328 (1.4)
Valve disease, *n* (%)	598 (5.0)	359 (3.0)	<0.0001	957 (4.0)
Aortic stenosis, *n* (%)	244 (2.1)	168 (1.4)	0.0002	412 (1.7)
Aortic regurgitation, *n* (%)	121 (1.0)	61 (0.5)	<0.0001	182 (0.8)
Mitral regurgitation, *n* (%)	219 (1.8)	107 (0.9)	<0.0001	326 (1.4)
Previous endocarditis, *n* (%)	64 (0.5)	61 (0.5)	0.79	125 (0.5)
Dilated cardiomyopathy, *n* (%)	392 (3.3)	237 (2.0)	<0.0001	629 (2.6)
Coronary artery disease, *n* (%)	1764 (14.9)	1278 (10.8)	<0.0001	3042 (12.8)
Previous MI, *n* (%)	330 (2.8)	227 (1.9)	<0.0001	557 (2.3)
Previous PCI, *n* (%)	300 (2.5)	243 (2.0)	0.01	543 (2.3)
Previous CABG, *n* (%)	48 (0.4)	26 (0.2)	0.01	74 (0.3)
Vascular disease, *n* (%)	2515 (21.2)	1286 (10.8)	<0.0001	3801 (16.0)
Atrial fibrillation, *n* (%)	0 (0.0)	0 (0.0)	-	0 (0.0)
Previous pacemaker or ICD, *n* (%)	372 (3.1)	257 (2.2)	<0.0001	629 (2.6)

Alcohol related diagnoses, *n* (%)	1349 (11.4)	1488 (12.5)	0.01	2837 (11.9)
Chronic kidney disease, *n* (%)	464 (3.9)	526 (4.4)	0.04	990 (4.2)
Lung disease, *n* (%)	2012 (16.9)	2102 (17.7)	0.12	4114 (17.3)
Sleep apnea syndrome, *n* (%)	392 (3.3)	409 (3.4)	0.54	801 (3.4)
COPD, *n* (%)	627 (5.3)	661 (5.6)	0.33	1288 (5.4)
Liver disease, *n* (%)	595 (5.0)	654 (5.5)	0.09	1249 (5.3)
Gastroesophageal reflux, *n* (%)	150 (1.3)	186 (1.6)	0.05	336 (1.4)
Thyroid diseases, *n* (%)	573 (4.8)	644 (5.4)	0.04	1217 (5.1)
Inflammatory disease, *n* (%)	455 (3.8)	520 (4.4)	0.03	975 (4.1)
Anemia, *n* (%)	1074 (9.0)	1193 (10.0)	0.01	2267 (9.5)
Previous cancer, *n* (%)	1466 (12.3)	1660 (14.0)	0.0002	3126 (13.2)
Poor nutrition, *n* (%)	868 (7.3)	961 (8.1)	0.02	1829 (7.7)
Cognitive impairment, *n* (%)	1418 (11.9)	1419 (12.0)	0.98	2837 (11.9)
Illicit drug use, *n* (%)	52 (0.4)	69 (0.6)	0.12	121 (0.5)

Values are n (%) or mean±SD. ICH = intracranial hemorrhage; SD = standard deviation; MI: myocardial infarction; PCI: percutaneous coronary intervention; CABG: coronary artery bypass graft; ICD: implantable cardioverter-defibrillator; COPD: chronic obstructive pulmonary disease;.

Table S3. Competing risk analyses: sub-distribution hazard ratios (sHR) (95% CI) associated with incident outcomes in non-traumatic intracranial hemorrhage with no AF (versus ischemic stroke survivors).

	**sHR**
Cardiovascular death	2.66 (2.52–2.80[a]
Non-cardiovascular death	0.92 (0.88–0.96)[b]
Myocardial infarction	0.55 (0.48–0.64)[c]
New onset heart failure	0.58 (0.54–0.63)[d]
Major bleeding	0.62 (0.59–0.66)[e]
New type of cerebrovascular event	1.20 (1.08–1.34)[f]

[a] Fine and Gray model for competing risks of cardiovascular and non-cardiovascular death; [b] Fine and Gray model for competing risks of non-cardiovascular and cardiovascular death; [c] Fine and Gray model for competing risks of myocardial infarction and all-cause death; [d] Fine and Gray model for competing risks of heart failure and all-cause death; [e] Fine and Gray model for competing risks of major bleeding and all-cause death; [f] Fine and Gray model for competing risks of new type of cerebrovascular and all-cause death.

Table S4. Baseline characteristics of matched patients with history of atrial fibrillation at baseline according to history of ischemic stroke or non-traumatic intracranial hemorrhage.

	**Ischemic Stroke**	**Non-Traumatic ICrH**	***p***	**Total**
	**(*n* = 3574)**	**(*n* = 3574)**		**(*n* = 7148)**
Age (years), mean±SD	79.8±9.7	79.4±9.4	0.13	79.6±9.5
Sex (male), *n* (%)	1889 (52.9)	1941 (54.3)	0.22	3830 (53.6)
Hypertension, *n* (%)	2700 (75.5)	2739 (76.6)	0.28	5439 (76.1)
Diabetes mellitus, *n* (%)	837 (23.4)	902 (25.2)	0.07	1739 (24.3)
Smoker, *n* (%)	190 (5.3)	227 (6.4)	0.06	417 (5.8)
Dyslipidemia, *n* (%)	867 (24.3)	916 (25.6)	0.18	1783 (25.0)
Obesity, *n* (%)	422 (11.8)	490 (13.7)	0.02	912 (12.8)
Heart failure, *n* (%)	1498 (41.9)	1413 (39.5)	0.04	2911 (40.7)
History of pulmonary edema, *n* (%)	119 (3.3)	100 (2.8)	0.19	219 (3.1)
Valve disease, *n* (%)	566 (15.8)	501 (14.0)	0.03	1067 (14.9)
Aortic stenosis, *n* (%)	220 (6.2)	183 (5.1)	0.06	403 (5.6)
Aortic regurgitation, *n* (%)	116 (3.2)	111 (3.1)	0.74	227 (3.2)

Mitral regurgitation, *n* (%)	257 (7.2)	233 (6.5)	0.26	490 (6.9)
Previous endocarditis, *n* (%)	33 (0.9)	57 (1.6)	0.01	90 (1.3)
Dilated cardiomyopathy, *n* (%)	359 (10.0)	293 (8.2)	0.01	652 (9.1)
Coronary artery disease, *n* (%)	967 (27.1)	924 (25.9)	0.25	1891 (26.5)
Previous MI, *n* (%)	155 (4.3)	134 (3.7)	0.21	289 (4.0)
Previous PCI, *n* (%)	135 (3.8)	131 (3.7)	0.8	266 (3.7)
Previous CABG, *n* (%)	43 (1.2)	34 (1.0)	0.3	77 (1.1)
Vascular disease, *n* (%)	982 (27.5)	720 (20.1)	<0.0001	1702 (23.8)
Atrial fibrillation, *n* (%)	3574 (100.0)	3574 (100.0)	-	7148 (100.0)
Previous pacemaker or ICD, *n* (%)	464 (13.0)	432 (12.1)	0.25	896 (12.5)
Alcohol related diagnoses, *n* (%)	247 (6.9)	285 (8.0)	0.09	532 (7.4)
Chronic kidney disease, *n* (%)	299 (8.4)	355 (9.9)	0.02	654 (9.2)
Lung disease, *n* (%)	799 (22.4)	880 (24.6)	0.02	1679 (23.5)
Sleep apnea syndrome, *n* (%)	202 (5.7)	233 (6.5)	0.13	435 (6.1)
COPD, *n* (%)	338 (9.5)	370 (10.4)	0.21	708 (9.9)
Liver disease, *n* (%)	172 (4.8)	184 (5.1)	0.51	356 (5.0)
Gastroesophageal reflux, *n* (%)	44 (1.2)	51 (1.4)	0.47	95 (1.3)
Thyroid diseases, *n* (%)	493 (13.8)	524 (14.7)	0.29	1017 (14.2)
Inflammatory disease, *n* (%)	206 (5.8)	244 (6.8)	0.06	450 (6.3)
Anemia, *n* (%)	515 (14.4)	592 (16.6)	0.01	1107 (15.5)
Previous cancer, *n* (%)	523 (14.6)	540 (15.1)	0.57	1063 (14.9)
Poor nutrition, *n* (%)	421 (11.8)	461 (12.9)	0.15	882 (12.3)
Cognitive impairment, *n* (%)	561 (15.7)	581 (16.3)	0.52	1142 (16.0)
Illicit drug use, *n* (%)	5 (0.1)	5 (0.1)	1	10 (0.1)

Values are n (%) or mean±SD. ICrH = non-traumatic intracranial hemorrhage; SD = standard deviation; MI: myocardial infarction; PCI: percutaneous coronary intervention; CABG: coronary artery bypass graft; ICD: implantable cardioverter-defibrillator; COPD: chronic obstructive pulmonary disease;

Table S5. Competing risk analyses: sub-distribution hazard ratios (sHR) (95% CI) associated with incident outcomes in non-traumatic intracranial hemorrhage with atrial fibrillation (versus ischemic stroke survivors).

	**sHR**
Cardiovascular death	1.95 (1.81–2.11)[a]
Non-cardiovascular death	0.84 (0.78–0.90)[b]
Myocardial infarction	0.71 (0.54–0.94)[c]
New onset heart failure	0.66 (0.59–0.74)[d]
Major bleeding	0.57 (0.52–0.64)[e]
New type of cerebrovascular event	1.53 (1.28–1.82)[f]

[a] Fine and Gray model for competing risks of cardiovascular and non-cardiovascular death; [b] Fine and Gray model for competing risks of non-cardiovascular and cardiovascular death; [c] Fine and Gray model for competing risks of myocardial infarction and all-cause death; [d] Fine and Gray model for competing risks of heart failure and all-cause death; [e] Fine and Gray model for competing risks of major bleeding and all-cause death; [f] Fine and Gray model for competing risks of new type of cerebrovascular and all-cause death.

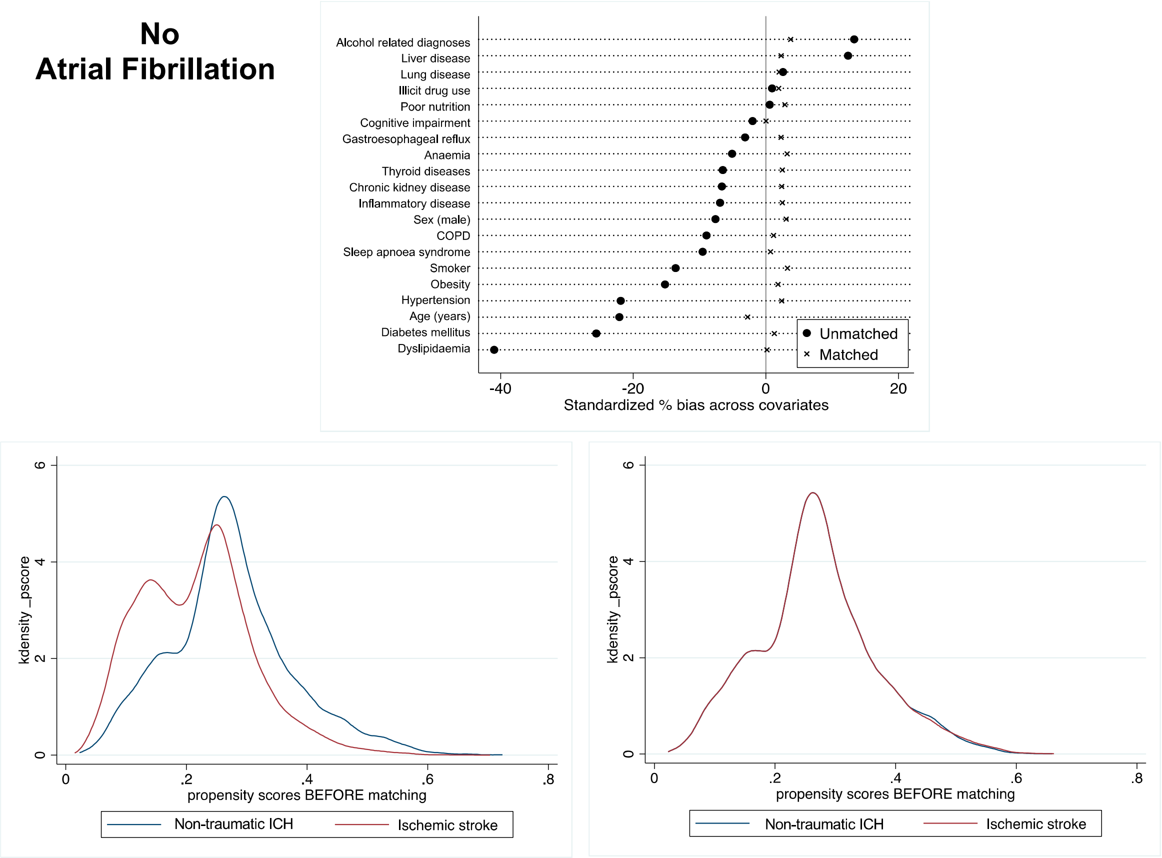

Figure S1. Propensity score matching in patients with no atrial fibrillation. Top panel: Standardized percentages of bias across main baseline characteristics in unmatched and matched patients with history of ischemic stroke and history of non-traumatic intracranial hemorrhage. Propensity score distribution for unmatched (left lower panel) and matched (right lower panel) populations of patients with history of ischemic stroke and history of non-traumatic intracranial hemorrhage.

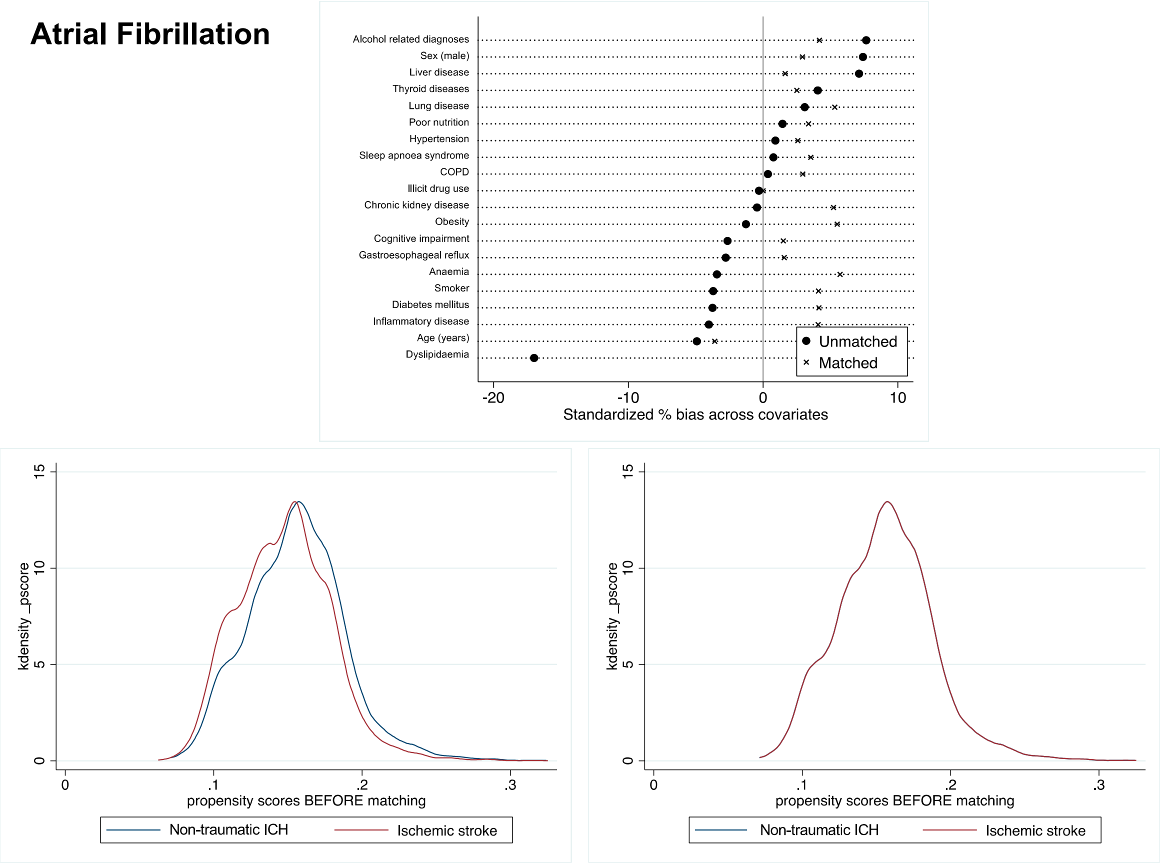

Figure S2. Propensity score matching in patients with atrial fibrillation. Top panel: Standardized percentages of bias across main baseline characteristics in unmatched and matched patients with history of ischemic stroke and history of non-traumatic intracranial hemorrrhae. Propensity score distribution for unmatched (left lower panel) and matched (right lower panel) populations of patients with history of ischemic stroke and history of non-traumatic intracranial hemorrhage.

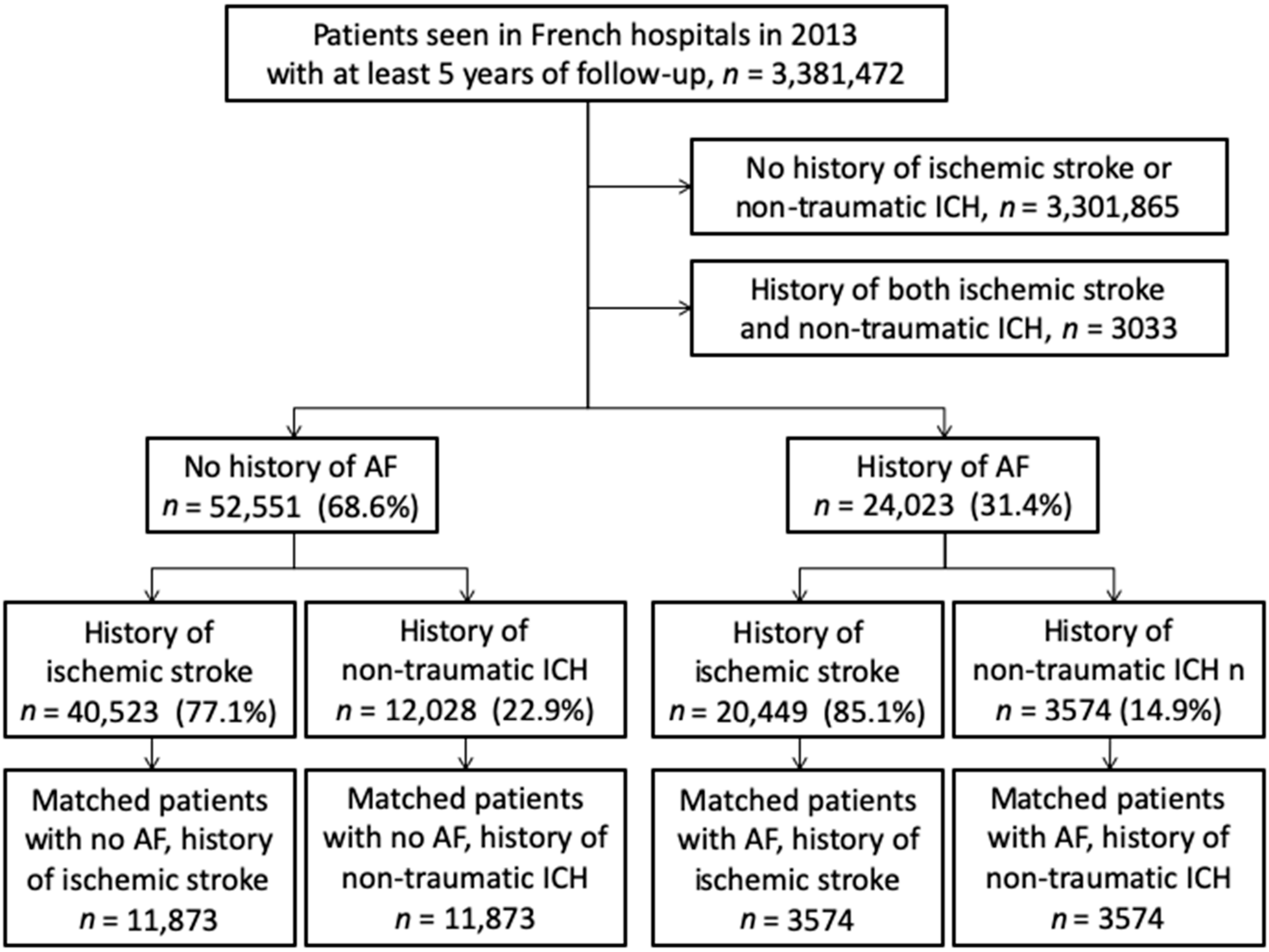

Figure S3. Flow chart of the study population.
